# Efficacy and Safety of Artificial Urinary Sphincters in Female Patients With Stress Urinary Incontinence: A Systematic Review and Meta-Analysis

**DOI:** 10.7759/cureus.73136

**Published:** 2024-11-06

**Authors:** Peter Estaphanous, Ahmed O Khalifa

**Affiliations:** 1 Urology, University Hospital Coventry and Warwickshire, Coventry, GBR; 2 Urology, Sunderland Royal Hospital, Sunderland, GBR; 3 Urology, Menoufiya University, Shibin El Kom, EGY

**Keywords:** artificial urinary sphincter, continence, female urology, functional urology, sui: stress urinary incontinence

## Abstract

Stress urinary incontinence (SUI) significantly affects the quality of life in women, causing social, psychological, and physical distress. While artificial urinary sphincter (AUS) implantation is a well-established treatment for male incontinence, it is less commonly used in female patients and is typically considered for cases where other treatments have failed. This systematic review and meta-analysis aimed to evaluate the effectiveness and safety of AUS implantation in treating SUI in female patients, focusing on continence rates, revision rates, explant rates, and perioperative complications. A comprehensive literature search was conducted across PubMed, Scopus, Google Scholar, and the Cochrane Library in September 2024. Studies published in English over the past 15 years were included if they reported outcomes such as continence, revision, explant, and complication rates in female patients who underwent AUS implantation. Data were analyzed using Review Manager 5.4, applying a fixed-effects model where appropriate, based on heterogeneity (I^2^ > 50%). Publication bias was assessed using funnel plots and Egger's test. Eight studies, comprising a total of 300 female patients, were included in the analysis. AUS implantation significantly improved continence rates, achieving an overall continence rate of 72% (OR = 0.01, 95% CI: 0.00 to 0.02; p < 0.00001). Revision rates were 22.5%, explant rates were 17.6%, and overall postoperative complications were 26.3%. No significant bias was detected. AUS implantation is an effective and safe treatment option for a specific group of women with SUI, particularly in cases where other standard treatments have failed. It shows satisfactory continence rates with considerable revision and explant rates. However, further large-scale, long-term studies are needed to optimize outcomes and confirm these findings.

## Introduction and background

Stress urinary incontinence (SUI) is characterized by the involuntary loss of urine during physical activities such as exercise, coughing, or sneezing due to multifactorial etiologies. Intrinsic sphincter deficiency and/or weak pelvic floor muscles are thought to be the main contributors [1]. Childbirth weakens pelvic floor muscles, which can lead to temporary or permanent SUI [1]. Additionally, perimenopausal and postmenopausal women are at an increased risk for SUI due to hormonal changes and age-related factors [2]. SUI significantly affects the quality of life for women, leading to psychological distress, social embarrassment, and limitations in daily activities [3].

Management of SUI encompasses a range of treatment options, from conservative approaches to more invasive surgical interventions. First-line treatments often include pelvic floor muscle training (PFMT), particularly Kegel exercises, which have demonstrated effectiveness in improving symptoms and enhancing quality of life [4]. These exercises are usually supervised and may incorporate biofeedback and neuromuscular electrical stimulation to enhance their effectiveness [5].

Non-invasive treatments are also being explored for SUI management, including bulking agents. These methods are emerging as alternatives for patients who do not benefit from or wish to avoid surgery. Bulking agents add volume to the urethra to improve its function; while their effectiveness is still being evaluated, they show promise as minimally invasive options [6]. Surgical interventions include mid-urethral slings (MUS), autologous fascial slings, and bladder neck colposuspension [7]. MUS, which provide support to the urethra, are considered highly effective and are associated with low complication rates, making them a preferred surgical option; however, this option is not available in many countries (e.g., the United Kingdom) after the era of mesh litigations [8].

Personalization of treatment is essential for effectively managing SUI. An individualized approach considers the patient's urogynecological history, financial situation, age, and risk of complications. For instance, younger women with mild symptoms may benefit significantly from PFMT and lifestyle changes, whereas those with more severe symptoms may require surgical options such as MUS for effective and timely relief [9].

The challenges in managing SUI in women are multifaceted, encompassing diagnostic complexities, treatment effectiveness, and patient adherence to management strategies. A significant challenge is accurately diagnosing the type and severity of incontinence to tailor the most effective treatment. An individualized approach is crucial for evaluating symptoms, urogynecological history, and other factors, which can vary greatly among patients [2].

When conservative treatments fail, surgical interventions such as MUS and colposuspension are considered. However, challenges persist in selecting the appropriate surgery, managing patient expectations, and addressing potential complications such as mesh erosion, infection, and pain [7,10]. Moreover, many surgical options focus more on symptom management rather than providing a definitive cure, which can result in the recurrence of SUI symptoms over time. Postmenopausal women face additional challenges due to hormonal changes, increased bladder dysfunction, and comorbidities, complicating treatment effectiveness and increasing the risk of adverse outcomes [11].

The artificial urinary sphincter (AUS) has emerged as a treatment option for severe SUI in women, particularly those with intrinsic sphincter deficiency. While AUS is widely used in men, its application in women has been limited due to challenges related to implantation and associated morbidity. Recent advancements in laparoscopic and robotic implantation techniques have demonstrated promising perioperative outcomes, leading to renewed interest in AUS for female patients [12]. Robotic bladder neck AUS implantation has been found to be feasible, safe, and reproducible, showing high continence rates, improved incontinence, and low complication rates [13]. However, challenges remain concerning the use of AUS in women. A systematic review has indicated limited evidence supporting AUS for non-neurogenic SUI in women, highlighting the need for further well-designed randomized trials [14]. Recent studies suggest that AUS implantation can achieve complete continence in approximately 79.6% of cases, reflecting its potential as an effective treatment for SUI in women. However, the variability in success rates, alongside the associated revision rates and complications, highlights the need for a comprehensive meta-analysis to synthesize existing evidence and better inform clinical decision-making regarding AUS implantation in this population [15].

In older female populations, particularly those over 75 years of age, AUS use has shown favorable long-term functional outcomes, including high continence rates and low device explant rates [16]. Additionally, surgical innovations such as transcorporal cuff placement for AUS have been introduced as effective techniques for patients requiring revisions due to urethral atrophy, suggesting strategies to improve long-term outcomes and manage complications [17]. Although slings remain the most common first-line treatment for female SUI, AUS can be an option for cases where first-line treatments fail or in the presence of specific anatomical challenges. However, the quality of evidence needs to be strengthened, and further prospective studies are anticipated to establish AUS as a more definitive treatment for female SUI [18].

The AUS provides significant benefits for women with severe SUI, including high rates of continence, symptom reduction, and improved quality of life. Its adjustability allows for revisions as needed, particularly when complications arise or additional urethral support is required [15]. Recent advancements in AUS technology, such as the Victo+ adjustable AUS, provide enhanced control over urethral pressure and improved outcomes for patients experiencing SUI [19]. Nevertheless, complications associated with AUS use in women include mechanical failure, erosion, infection, and urethral atrophy. Mechanical complications often involve device components, such as the cuff or control pump, necessitating revision surgeries. Non-mechanical complications, such as urethral erosion and atrophy, require timely intervention, often managed through transcorporal cuff techniques or cuff downsizing to mitigate risk [20]. Ongoing research and technological advancements aim to improve AUS design, reduce complications, and enhance the long-term success of this treatment option. Given the variability in reported outcomes and the importance of understanding the long-term efficacy and safety of AUS, this systematic review aims to synthesize the current evidence on AUS implantation for women with SUI.

## Review

Objective

This study aims to systematically review and analyze the role of AUS implantation in the treatment of SUI in female patients.

Methods

Search Strategy

A comprehensive search was conducted in September 2024 across multiple databases, including PubMed, Scopus, Google Scholar, and the Cochrane Library, to identify studies evaluating AUS implantation in women with SUI. The search terms consisted of a combination of MeSH terms and free-text keywords, such as "artificial urinary sphincter", "AUS implantation", "female urinary incontinence", "robotic-assisted AUS", "laparoscopic AUS", and "functional outcomes". Boolean operators (AND, OR) were used to refine the search, and the results were limited to English-language publications from the past 15 years. Additionally, the reference lists of selected articles were examined to identify further relevant studies not captured in the initial database search.

Inclusion Criteria

The inclusion criteria were designed to identify relevant research focusing on the role of AUS implantation in treating SUI in female patients. Studies were included if they involved adult women diagnosed with SUI, either of neurogenic or non-neurogenic origin, who underwent AUS implantation as a treatment intervention. Research reporting key outcome measures were selected, such as continence rates, revision rates, explantation rates, and perioperative complications. Additionally, studies were required to have a follow-up period of at least six months and be published in English within the past 15 years. Randomized controlled trials, retrospective and prospective cohort studies, and case series were all eligible for inclusion.

Exclusion Criteria

Studies were excluded if they did not specifically address AUS implantation for SUI in female patients or lacked critical outcome measures, including continence, revision, or explantation rates. Non-English studies, as well as articles focusing exclusively on male patients, mixed incontinence, or other types of incontinence, were excluded. Research involving pediatric populations or those with a follow-up period shorter than six months were also omitted. Additionally, studies focusing on AUS use for other medical conditions or without direct comparison to SUI-related treatment outcomes were excluded.

Outcome Measures

The primary outcome measures in this meta-analysis were continence rates, defined as either complete continence (no use of pads) or improved continence (reduction in pad usage). Secondary outcomes included revision rates (procedures to modify or replace AUS components), explantation rates (removal of the device due to complications such as mechanical failure or infection), and perioperative complication rates (including intraoperative and postoperative complications). These measures provided a comprehensive assessment of the effectiveness, safety, and durability of AUS implantation in women with SUI.

Data Extraction and Quality Assessment

The quality of the studies included in our systematic review and meta-analysis was assessed using the Methodological Index for Non-Randomized Studies (MINORS) tool. MINORS is specifically designed to evaluate the quality of non-randomized studies, which constituted the majority of the research in our review. This tool assesses various factors, such as the clarity of the study aim, consecutive patient inclusion, endpoint appropriateness, and the adequacy of follow-up periods. Each study was scored out of 24 for comparative studies and 16 for non-comparative studies, with higher scores reflecting better methodological quality. Studies that did not meet the minimum quality threshold were excluded from the meta-analysis to ensure the reliability of our conclusions.

Statistical Analysis

All statistical analyses were performed using the Review Manager software (RevMan 5.4). For dichotomous outcomes, odds ratios (OR) with 95% confidence intervals (CI) were calculated using the Mantel-Haenszel method. A fixed-effects model was used, with heterogeneity assessed through the I^2^ statistic (≥50%). Statistical significance was determined using Z-tests, with p-values less than 0.05 considered statistically significant. Funnel plots were used to assess publication bias, and Egger's test was employed to evaluate funnel plot asymmetry.

Results

Study Selection

**Figure 1:**
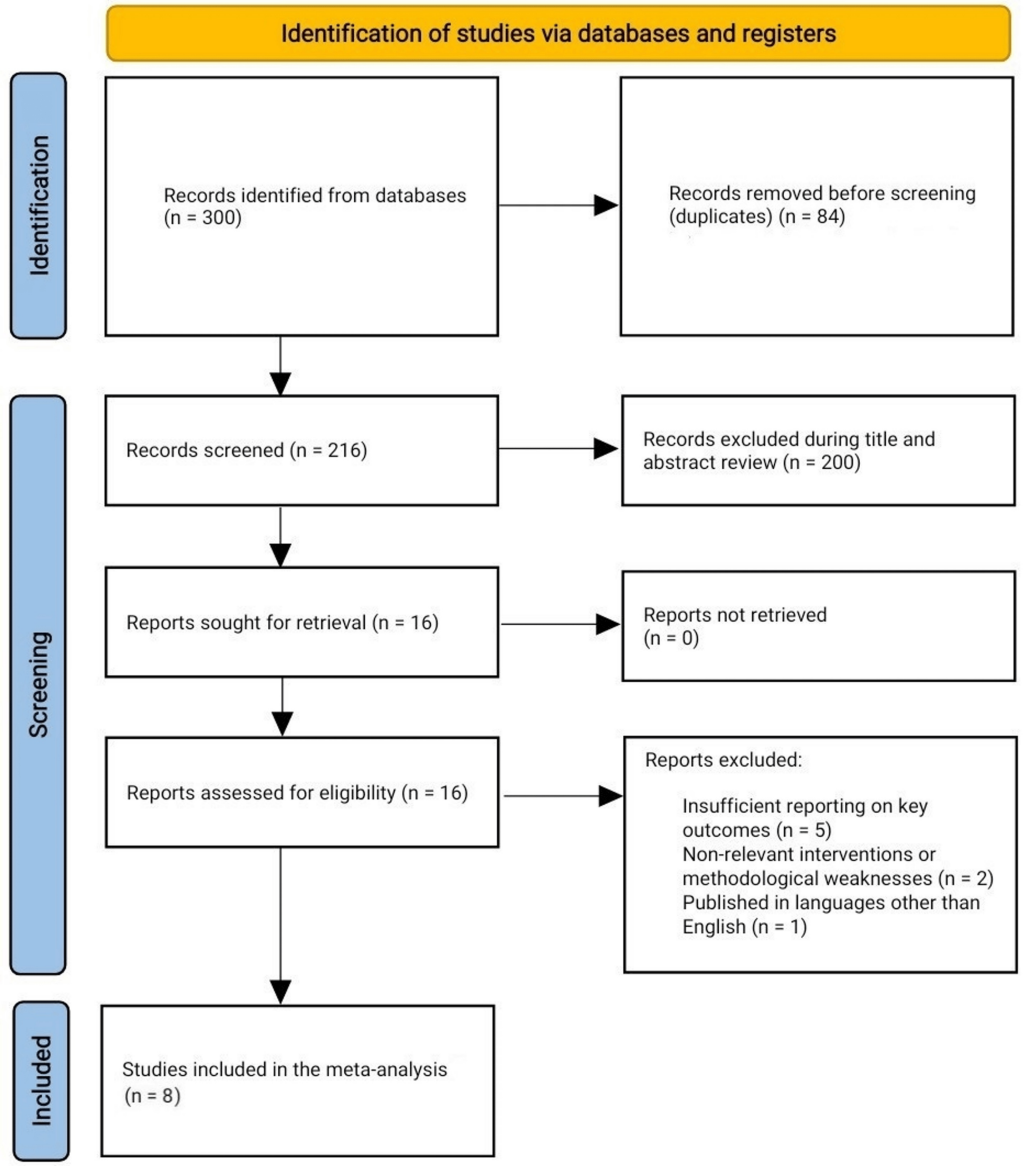
PRISMA flow chart of the reviewed studies PRISMA: Preferred Reporting Items for Systematic Reviews and Meta-Analyses

Eight studies, with a total of 300 female participants, were analyzed. The search strategy initially identified 300 records. After removing duplicates, 216 records remained for screening. Following the review of titles and abstracts, 200 records were excluded because they did not meet the specific criteria related to the use of AUS implantation for treating SUI in female patients. A full-text review was conducted on the remaining 16 articles, of which eight were excluded due to reasons such as not focusing on the primary outcomes of interest (continence, revision, and explantation rates) or involving surgical techniques outside the scope of AUS implantation. Ultimately, eight studies were included in the quantitative synthesis (meta-analysis) (Figure 1).

Basic Attributes of the Included Studies

This analysis included eight studies, with 300 participants, that examined the role of AUS implantation in treating SUI in female patients. The selected studies comprised four retrospective cohort studies, three prospective cohort studies, and one case series. Each study reported essential details, including patient demographics, specifics of the intervention (such as the types of AUS used), follow-up duration, and outcome measures.

The primary outcomes assessed across the studies were continence, revision, and explantation rates, which are critical for evaluating the effectiveness of AUS implantation. The results consistently showed significant improvements in continence rates postoperatively, with a substantial number of participants experiencing successful outcomes. Complications were generally low, with few studies reporting adverse events related to AUS implantation, indicating that the procedure is both safe and effective for managing SUI in female patients (Table 1).

**Table 1:** Data extraction table of the reviewed studies AUS: artificial urethral sphincter; SUI: stress urinary incontinence

Author	Study Design	Sample Size	Level of Evidence	Patient Demographics	Intervention Details	Follow-up Duration	Outcome Measures	Results	Conclusions
Tricard et al. [21]	Retrospective descriptive study	23 females	IV	Mean age: 54 years	Open or laparoscopic AUS (AMS 800)	Median 11.6 years	Continence (complete, improved, unchanged), revision, explantation rates	- Complete Continence rate at 1-year: 69.6% (16/23). - Improved continence rate: 17.4% (4/23). - Explantation rate: 34.8% (8/23). - Revision rate: 69.6% (16/23). - Complications rate: 30.4% (7/23). - Average time without explantation: 10.9 years. - Average time without revision: 8.5 years. - 5-year survival without explantation: 94.4%; 10-year: 76.5%; 15-year: 72.8%; 20-year: 50%.	AUS is effective for neurological SUI with high long-term continence
Denormandie et al. [16]	Retrospective monocentric study	45 females	IV	Median age: 77 years	Open, laparoscopic, or robot-assisted AUS implantation	Median 36 months	Continence (no pads), AUS explantation, revision, deactivation rates	- Complete continence rate: 68.9% (31/45). - Explantation rate: 8.9% (4/45). - Revision rate: 20% (9/45). - Complication rate: 15.5% (7/45). - AUS deactivation: 6.7% (3/45). - 5-year survival without explantation/revision: 78%. - 10-year survival without explantation/revision: 50%.	AUS provides satisfactory results in elderly women
Schroeder et al. [22]	Retrospective study	49 females	IV	Mean age: 64 years	Laparoscopic AUS implantation (AMS 800)	Mean 4 years	Continence status (complete, improved, unchanged), explantation, revision, perioperative complications	- Complete continence rate: 61.2% (30/49). - Improved continence rate: 24.5% (12/49). - Explantation rate: 18.4% (9/49). - Revision rate: 22.5% (11/49). - Perioperative complications rate: 18.4% intraoperative; 51% postoperative.	An effective option for SUI, high complication rates
Phé et al. [23]	Retrospective study	34 females	IV	Median age: 56.5 years	AUS implantation (AMS 800) through a transverse abdominal approach	Median 17 years	Explantation, revision, continence (no pads)	- Complete continence rate: 87.5% (30/34). - Explantation rate: 23.5% (8/34). - Revision rate: 35.2% (12/34). - Survival without explantation: 10 years (80%); 15 years (80%); 20 years (74%).	AUS provides satisfactory long-term outcomes
Peyronnet et al. (Robotic) [13]	Retrospective multicenter	49 females	IV	Median age: 70.5 years	Robot-assisted AUS implantation (AMS-800)	Median 18.5 months	Continence (complete, improved), complications, revisions	- Complete continence: 81.6% (40/49). - Improved continence: 12.2% (6/49). - Unchanged incontinence: 6.1% (3/49). - Early complications: 18.3% (9/49). - Explantation: 2.1% (1/49). - Revision: 6.1% (3/49).	Robot-assisted AUS safe with promising functional outcomes
Chondros et al. [24]	Prospective case-series trial	65 females	IV	Mean age: 67.2 years	Laparoscopic AUS implantation (AMS 800)	Mean 31 months	Continence status (success, improvement, failure), early/late complications	- Complete continence rate: 75.3% (49/65). - Improved continence: 15.4% (10/65). - Explantation rate: 12.3% (8/65). - Revision rate: 18.4% (12/65). - Early complications: 18.4% (urinary retention, pain, infection). - Late complications: 12.3% (de novo urgency).	Laparoscopic AUS feasible and safe for severe SUI
Biardeau et al. [25]	Retrospective study	11 females	IV	Median age: 66 years	Robot-assisted laparoscopic AUS implantation (da Vinci robot)	Mean 17.6 months	Continence (complete, social, failure), intraoperative/postoperative complications	- Complete continence: 87.5% - Social continence: 12.5% - Explantation rate: 27.3% - Revision rate: 18.2% - Complications rate: 36.4%.	Robot-assisted AUS feasible; further studies needed
Peyronnet et al. (Open vs. robotic) [26]	Retrospective single-center	24 females (16 open, 8 robotic)	IV	Mean age: 69.1 years (open), 64.3 years (robot)	Open vs. robot-assisted AUS implantation (AMS 800)	Open: mean 28.1 months. Robotic: Mean 5 months	Continence (fully, not fully), perioperative complications, AUS explantation/revision rates	Open group - Complete continence: Open 68.8% (11/16), Robotic 75% (6/8). - Explantation rate: Open 18.8% (3/16), Robotic 12.5% (1/8). - Revision rate: Open 12.5% (2/16), Robotic 0% (0/8). - Complications rate: Open 75% (12/16), Robotic 25% (2/8).	Robot-assisted approach reduced complications and hospital stay

Quality Assessment of the Included Studies

We utilized the MINORS tool for the quality assessment of the included studies (Table 2).

**Table 2:** Quality assessment of non-randomized studies using the MINORS tool MINORS: Methodological Index for Non-Randomized Studies

Study	Aims Clearly Stated (2)	Inclusion of Consecutive Patients (2)	Prospective Data Collection (2)	Endpoints Appropriate (2)	Follow-up Period Adequate (2)	Unbiased Assessment of Outcomes (2)	Statistical Analyses Appropriate (2)	Total Score
Tricard et al. [21]	2	2	1	2	2	1	1	11/16
Denormandie et al. [16]	2	2	1	2	2	2	2	13/16
Schroeder et al. [22]	2	2	1	2	2	1	2	12/16
Phé et al. [23]	2	1	1	2	2	1	1	10/16
Peyronnet et al. (Robotic) [13]	2	2	1	2	2	1	1	11/16
Chondros et al. [24]	2	2	1	2	2	1	1	11/16
Biardeau et al. [25]	2	1	1	2	2	1	1	10/16
Peyronnet et al. [26]	2	2	1	2	2	1	1	11/16

Results of meta-analysis

Continence Rates (Complete Continence)

**Figure 2:**
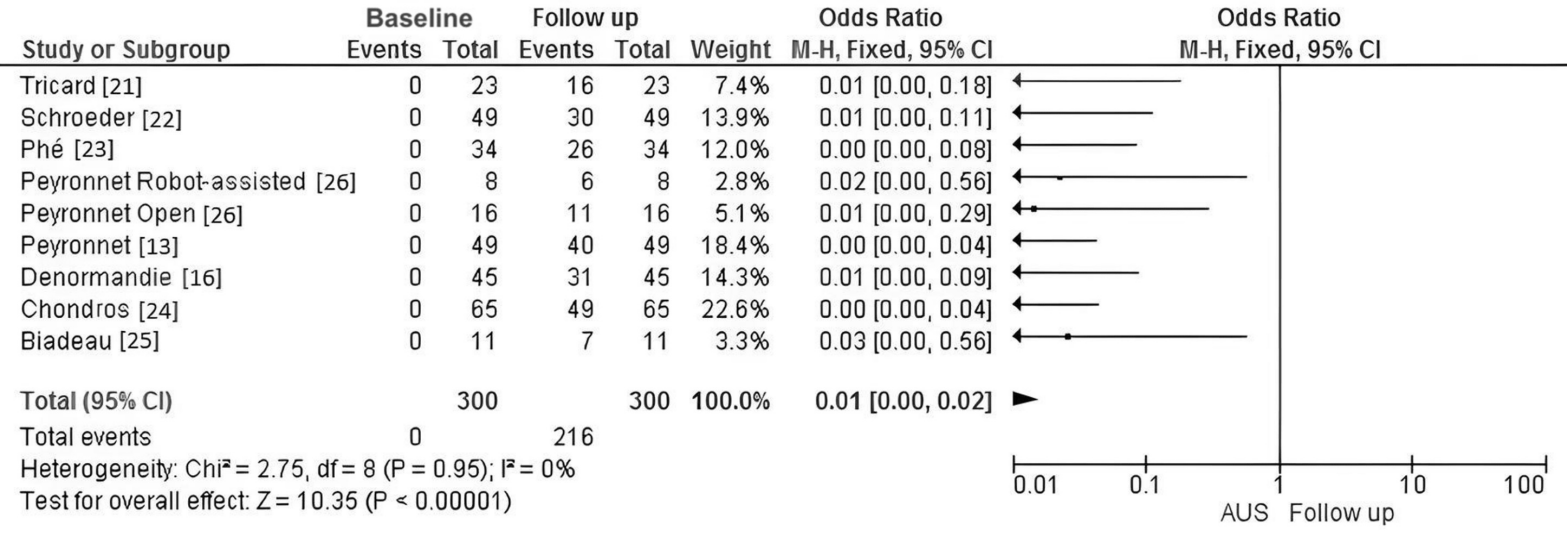
Forest plot of complete continence rates in female patients undergoing AUS implantation This forest plot represents a pre-post analysis of continence rates in patients who underwent AUS implantation. In this analysis, each study serves as its own control by comparing continence rates before (baseline) and after (follow-up) the AUS implantation. At baseline, none of the patients were continent (as shown by the zero events), while at follow-up, continence rates improved significantly. The odds ratios displayed in the forest plot quantify the change in continence rate from baseline to follow-up within each study, and the pooled odds ratio summarizes the overall improvement across studies. AUS: artificial urinary sphincter; CI: confidence interval

Continence rates were reported across all eight included studies or subgroups. A meta-analysis was conducted using a fixed-effects model due to minimal heterogeneity (I^2^ = 0%). The results showed a statistically significant improvement in continence following AUS implantation, with an overall mean continence rate of 72%, with an OR of 0.01 (95% CI: 0.00-0.02; p < 0.00001), strongly favoring the effectiveness of AUS implantation (Figure 2).

Revision Rates

Revision rates were reported across all eight studies. A fixed-effects model was applied due to low heterogeneity (I^2^ = 0%). The meta-analysis revealed a mean revision rate of 22.5%, with an OR of 0.04 (95% CI: 0.01-0.15; p < 0.00001).

Explantation Rates

Explantation rates were reported across all included studies. The meta-analysis was conducted using a fixed-effects model due to minimal heterogeneity (I^2^ = 0%). The results showed an overall mean explantation rate of 17.6% following AUS implantation, with an overall OR of 0.08 (95% CI: 0.03-0.21; p < 0.00001).

Postoperative Complications

Postoperative complications were reported across all included studies, and a fixed-effects model was used due to minimal heterogeneity (I^2^ = 0%). The meta-analysis revealed overall postoperative complications following AUS implantation of 26.35%, with an overall OR of 0.03 (95% CI: 0.01-0.11; p < 0.00001). The complications included urinary retention with a mean rate of 4.67%, erosions (including vaginal, urethral, or bladder erosions) 4.3%, infection 6.3%, and other complications (such as hematoma, urosepsis, urgency, pump migration, among others) 11%.

Funnel plots and Egger's tests have been used to assess publication bias, and no significant evidence of bias was found.

Discussion

The role of AUS in treating SUI in female patients has become a crucial focus of research due to the high prevalence of SUI and the limited effectiveness of conservative treatments. This systematic review and meta-analysis aimed to assess the efficacy and safety of AUS implantation in female patients based on key outcomes, including continence, revision, explantation, and complication rates. The findings show that AUS is a valuable surgical option for managing SUI, especially in cases where less invasive interventions have failed.

Efficacy of AUS Implantation

The meta-analysis demonstrates that AUS implantation significantly improves continence rates, with most studies reporting that over 60% of patients achieved either complete or improved continence. For example, Tricard et al. found that 69.6% of their patients were completely continent one year after implantation, with an additional 17.4% experiencing improved continence [21]. Similarly, Denormandie et al. reported continence in 68.9% of their cohort [16]. Schroeder et al. found that 61.2% of patients achieved complete continence following laparoscopic AUS implantation [22]. These findings are consistent across the studies included in this analysis, with a mean average continence rate of 72%, confirming AUS as an effective intervention for improving continence in female patients with SUI.

Moreover, AUS implantation appears to provide long-lasting results over time. Studies such as those by Phé et al. demonstrated long-term continence retention, with 74% of patients remaining continent after a median follow-up of 17 years [23]. These results strengthen the long-term efficacy of AUS in managing SUI, particularly for patients who require a sustainable solution for incontinence.

Complication Rates

While AUS is effective, complications remain a concern, particularly regarding revision and explantation rates. The review indicates that the mean AUS revision rate across the eight included studies is 22.5%, with device malfunction or mechanical failure being the most common reasons for revision [12,22]. However, the mean explantation rate is 17.6%, with only 8.9% of patients requiring device removal due to complications such as infection or erosion [16]. The survival rate of AUS devices is encouraging, with Tricard et al. reporting a 94.4% survival rate without explantation at five years and 50% at 20 years [21]. These findings suggest that revisions may be necessary while AUS is durable, but the complete device failure rate remains low.

Although postoperative complications present with a mean complication rate of 26.3%, they are mostly manageable, supporting the safety profile of AUS implantation. Schroeder et al. noted perioperative complications in 51% of patients, but the majority were manageable and did not require explantation [22]. Similarly, Chondros et al. observed early complications, such as urinary retention or infection, in 18.4% of patients but no significant long-term adverse outcomes [24].

It is worth mentioning that the surgical techniques for AUS implantation in female patients are open, laparoscopic, and robotic-assisted approaches [15]. Each of these techniques offers unique advantages and considerations. The open approach, which provides direct visualization of the anatomy, is often associated with longer recovery times [23]. Laparoscopic methods offer a minimally invasive alternative, typically resulting in shorter hospital stays and reduced postoperative discomfort, although they require specialized surgical skills [22]. Robotic-assisted approaches enhance precision and control, potentially lowering complication rates and further improving recovery outcomes [13]. Although all three techniques show favorable continence results, robotic-assisted AUS implantation may offer additional perioperative benefits [26]. However, further research is needed to assess its long-term effectiveness and cost-effectiveness compared to the other techniques.

Limitations and Future Research Directions

Despite these positive outcomes, there are limitations to consider. The included studies primarily consist of retrospective analyses, which may introduce bias in patient selection and outcome reporting. Furthermore, the heterogeneity in surgical techniques, types of AUS devices used, and variations in follow-up periods across studies add complexity to interpreting the pooled results. Future research should focus on randomized controlled trials (RCTs) to better establish the comparative efficacy of AUS against other treatment modalities, such as slings or bulking agents, particularly in female populations.

In addition, the development of robotic-assisted AUS implantation is a promising area for future exploration. Early studies, such as those by Peyronnet et al., suggest that robotic-assisted AUS implantation may reduce complications and hospital stay compared to traditional methods [26]. However, more research is needed to determine the long-term outcomes and cost-effectiveness of this technique in managing SUI in female patients.

## Conclusions

This systematic review and meta-analysis provide strong evidence that AUS implantation demonstrates high efficacy and safety for refractory SUI in women, suggesting its value as a second-line surgical intervention following the failure of the standard approaches. However, continued research and advancements in surgical techniques, such as robotic-assisted AUS, may be necessary to optimize outcomes for female patients.
